# Novel *CSF1-S100A10* fusion gene and *CSF1* transcript identified by RNA sequencing in tenosynovial giant cell tumors

**DOI:** 10.3892/ijo.2014.2326

**Published:** 2014-03-05

**Authors:** IOANNIS PANAGOPOULOS, PETTER BRANDAL, LUDMILA GORUNOVA, BODIL BJERKEHAGEN, SVERRE HEIM

**Affiliations:** 1 Section for Cancer Cytogenetics, Institute for Cancer Genetics and Informatics,; 2 Departments of Oncology, The Norwegian Radium Hospital, Oslo University Hospital;; 3 Pathology, The Norwegian Radium Hospital, Oslo University Hospital;; 4 Centre for Cancer Biomedicine, University of Oslo, Oslo, Norway; 5 Faculty of Medicine, University of Oslo, Oslo, Norway

**Keywords:** tenosynovial giant cell tumor, RNA sequencing, *CSF1*, *CSF1-S100A10* fusion gene, 3′-untranslated region

## Abstract

RNA-sequencing was performed on three tenosynovial giant cell tumors (TSGCT) in an attempt to elicit more information on the mechanisms of *CSF1* expression in this tumor type. A novel *CSF1-S100A10* fusion gene was found in a TSGCT that carried the translocation t(1;1)(q21;p11) as the sole karyotypic abnormality. In this fusion gene, the part of *CSF1* coding for the CSF1 protein (exons 1–8 in sequences with accession nos. NM_000757 and NM_172212) is fused to the 3′-part of *S100A10*. Since the stop codon TAG of *CSF1* is present in it, the *CSF1-S100A10* fusion gene’s predominant consequence seems to be the replacement of the 3′-untranslated region (UTR) of *CSF1* (exon 9; nt 2092–4234 in sequence with accession no. NM_000757 or nt 2092–2772 in NM_172212) by the 3′-end of *S100A10* (exon 3; nt 641–1055 in sequence with accession no. NM_002966). In the other two TSGCT, a novel *CSF1* transcript was detected, the same in both tumors. Similar to the occurrence in the *CSF1-S100A10* fusion gene, the novel *CSF1* transcript 3′-UTR is replaced by a new exon located ∼48 kb downstream of *CSF1* and 11 kb upstream of *AHCYL1*. Although only 3 TSGCT were available for study, the finding in all of them of a novel *CSF1-S100A10* fusion gene or *CSF1* transcript indicates the existence of a common pathogenetic theme in this tumor type: the replacement of the 3′-UTR of *CSF1* with other sequences.

## Introduction

Tenosynovial giant cell tumors (TSGCT) usually occur in tendon sheaths and in the synovia of joints and bursae (1). The tumor is also known under a variety of other names including pigmented villonodular synovitis (PVNS) (1,2). TSGCT is more common in women than in men, may occur at any age, and may be more or less diffuse in growth pattern (3). The localized tumors are the most common, they are found predominantly in fingers, develop slowly over years, and manifest themselves as small, circumscribed tumors that recur in 10–20% of the cases. Most diffuse TSGCT are larger than their localized counterparts, occur in large joints (in particular the knee), have an expansive growth pattern, and up to 50% recur locally. TSGCT are often moderately cellular with mononuclear cells, scattered giant cells, xanthoma cells and some degree of collagenization.

The pathogenetic mechanisms underlying TSGCT have been debated; some authors see them as inflammatory in nature (1,4), whereas the predominating view at present holds that they are neoplastic (2,3,5), something that also concurs with the opinion held by the original describers of the entity (6). The description of TSGCT-like lesions after injection of pro-inflammatory agents (7) and the finding of polyclonality in lyonization studies (8,9) argue for an inflammatory disease mechanism, whereas the finding of monoclonality in another X-inactivation study (10) as well as the detection of recurrent clonal chromosomal aberrations (11–15) suggest a neoplastic pathogenesis. Partly because of the uncertainty regarding the nature of this disease, the existence of malignant TSGCT has been questioned, but it seems that such tumors do exist, rare though they are (16–18). The preferred treatment for TSGCT is surgery with free margins to avoid recurrence, and the prognosis is good (3). At present, no adjuvant therapy is recommended for most lesions.

It seems that over 50% of TSGCT have an abnormal karyotype, but this can of course be a misrepresentation because there is a general tendency towards reporting abnormal findings more often than normal ones (19). Most TSGCT with chromosomal aberrations are near-diploid or pseudodiploid and ∼50% carry balanced chromosomal rearrangements. So far, 28 cases of the diffuse type and 18 localized TSGCT have been reported with chromosomal aberrations (http://cgap.nci.nih.gov/Chromosomes/Mitelman). Gains of chromosome 7 and/or chromosome 5 have been the most common numerical aberrations by karyotyping but only in the diffuse type of TSGCT. Structural rearrangements preferably involve chromosomal areas 1p11–13, 2q35–37, and 16q22–24 (14) and two distinct subgroups of TSGCT can be recognized based on the above-mentioned structural aberrations: those with 1p11–13 rearrangements and those with 16q22–24 rearrangements. Several translocation partners have participated in the changes affecting 1p11–13 (http://cgap.nci.nih.gov/Chromosomes/Mitelman) of which 2q35–37 is the most common (14). These results were confirmed in a study by West *et al* (20) who additionally identified the colony-stimulating factor-1 (*CSF1* or *M-CSF1*) locus at 1p13 as a molecular target of chromosomal rearrangements in 20 of 23 TSGCT. Chromosome 2 was the translocation partner in a subset (3 of 10) of tumors and collagen type VI α-3 (*COL6A3*) was then identified as the partner gene involved at 2q37. Moreover, combined interphase FISH and CSF1 immunohistochemistry demonstrated that only a minority (2–16%) of the cells in the tumor samples carried the t(1;2)(p13;q37) and that only those cells expressed *CSF1*, while tissue microarray analyses showed that the CSF1 receptor gene *CSF1R* is highly overexpressed in TSGCT (20). To explain these findings, it was suggested that the translocation involving *CSF1* and *COL6A3* results in a high level of *CSF1* expression in the neoplastic parenchyma cells, which in turn recruit also non-neoplastic *CSF1R*-expressing cells. In the words of West *et al* (20): ‘The *CSF1-COL6A3* translocation in TSGCT and PVNS is reminiscent of the translocation that defines DFSP (dermatofibrosarcoma protuberans). In this malignancy, t(17;22) brings *PDGF-B* under control of the strong *COL1A1* promoter. The posttranslationally processed form of the fusion protein is a fully functional PDGF-B protein that may stimulate oncogenesis through its receptor, PDGFRB. The *PDGFRB* receptor is also upregulated in DFSP, suggesting an autocrine loop’. Based on the suggestion that a fusion of these genes through the translocation would result in overexpression of *CSF1* due to a strong *COL6A3* promoter, Möller *et al* (21) performed RT-PCR on six TSGCT cases with t(1;2) to search for a putative *COL6A3-CSF1* fusion gene. Such fusion transcripts were detected in three cases and in one of them it was in-frame. In all cases, however, the breakpoints in *CSF1* appeared downstream of exon 5, indicating that the amino-terminal part of CSF1, which interacts with its receptor CSF1R, was not encoded by the identified chimeric transcripts (21,22). The authors concluded that ‘the *COL6A3-CSF1* fusion transcripts identified in the present study are unlikely to produce a functional protein containing the receptor-binding part of CSF1, which is required for the mechanism described earlier’ (21). In another study, Cupp *et al* (23) examined 57 TSGCT/PVNS and in all of them found expression of *CSF1* mRNA and/or CSF1 protein, including 22 tumors (39%) that lacked a *CSF1* translocation, suggesting that alternative mechanisms may lead to upregulation of *CSF1*.

In the present study we performed RNA-sequencing of three TSGCT in an attempt to elicit more information on the mechanisms of altered *CSF1* expression in this tumor type.

## Materials and methods

### Ethics statement

The study was approved by the regional ethics committee (Regional komité for medisinsk forskningsetikk Sør-Øst, Norge, http://helseforskning.etikkom.no), and written informed consent was obtained from the patients.

### Patients

Case 1 was a localized TSGCT. Cases 2 and 3 were both diffuse type tumors and were previously reported (11). Culturing and cytogenetic analysis were done as previously described (11).

### High-throughput paired-end RNA-sequencing

Total RNA was extracted from the three tumors using TRIzol reagent according to the manufacturer’s instructions (Invitrogen, Life Technologies, Oslo, Norway) and its quality was checked by Experion (Bio-Rad Laboratories, Oslo, Norway). Total RNA (3 *μ*g) was sent for high-throughput paired-end RNA-sequencing to the Norwegian Sequencing Centre at Ullevål Hospital (http://www.sequencing.uio.no/). The Illumina software pipeline was used to process image data into raw sequencing data and only sequence reads marked as ‘passed filtering’ were used in the downstream data analysis. A total of 60 million reads were obtained. The FASTQC software was used for the quality control of the raw sequence data (http://www.bioinformatics.babraham.ac.uk/projects/fastqc/). The software FusionMap and the associated pre-built Human B37 and RefGene from the FusionMap website were used for the discovery of fusion transcripts (24), release date 2012-04-16 (http://www.omicsoft.com/fusionmap/).

### PCR and 3′-RACE

The primers used for PCR amplification and sequencing are listed in Table I. The Human Universal Reference Total RNA was used as control (Clontech Laboratories, Takara Bio Group, France). According to the company’s information it is a ‘mixture of total RNAs from a collection of adult human tissues, chosen to represent a broad range of expressed genes. Both male and female donors are represented’. Expression analysis was performed using the human cell line MTC panel (Clontech Laboratories, Takara Bio Group), which contains cDNA from the cell lines Ad5, SKOV-3, Saos2, A431, Du145, H1299, HeLa, and MCF7.

Total RNA (2 *μ*g) was reverse-transcribed in a 20 *μ*l reaction volume using iScript Advanced cDNA Synthesis kit for RT-qPCR according to the manufacturer’s instructions (Bio-Rad Laboratories, Oslo, Norway). The cDNA was diluted to 20 ng equivalent of RNA/*μ*l and 2 *μ*l were used as templates in subsequent PCR assays. The 25-*μ*l PCR volume contained 12.5 *μ*l of Premix Taq (Takara-Bio), 2 *μ*l of diluted cDNA (or 2 *μ*l of cDNA from the cDNA panels which corresponds to 4 ng of cDNA), and 0.4 *μ*M of each of the forward and reverse primers. The PCRs were run on a C-1000 Thermal cycler (Bio-Rad Laboratories). The PCR conditions were: an initial denaturation at 94˚C for 30 sec followed by 35 cycles of 7 sec at 98˚C, 120 sec at 68˚C and a final extension for 5 min at 68˚C.

For detection of the *CSF1-S100A10* fusion transcript the primer set CSF1-1886F/S100A10-840R was used. For the amplification of the new *CSF1* transcript (transcript 5, see below) the primer set CSF1-1886F/CSF1-3end-R1out was used. For the amplification of *CSF1* transcript 1 (NM_000757) the primer set CSF1-1886F/CSF1-2176R was used. This primer combination and the primer set CSF1-2057F and CSF1-trans1-2123R (which is used for real-time PCR) also detect the transcript variant 2 (NM_172210) which lacks an alternate in-frame segment in exon 6 compared to transcript 1, resulting in a shorter isoform b compared to isoform a. No investigation was done for the expression of transcript 2, and for simplicity we refer in this report to primer set CSF1-1886F/CSF1-2176R as amplifying transcript 1 (in reality 1 and 2). For the amplification of *CSF1* transcript 4 (NM_172212), the primer set CSF1-1886F/CSF1-trans4-2130R was used. For amplification of *S100A10* wild-type transcript, the primer set S100A10-555F/S100A10-840R was used.

For 3′-RACE, 1 *μ*g of total RNA was reverse-transcribed in a 20 *μ*l reaction volume with the A3RNV-RACE primer (Table I) using iScript Select cDNA Synthesis kit according to the manufacturer’s instructions (Bio-Rad Laboratories). A 1 *μ*l template was amplified using the outer primer combination CSF1-1886F/A3R-1New. The amplified products were diluted 1:1,000 and 1 *μ*l of the diluted amplified PCR product was used as template in nested PCR with the primers CSF1-2006F/A3R-2New. For both PCRs the 25 *μ*l reaction volume contained 12.5 *μ*l of Premix Taq (Takara-Bio), template, and 0.4 *μ*M of each of the forward and reverse primers. PCR cycling consisted of an initial step of denaturation at 94˚C for 30 sec followed by 35 cycles of 7 sec at 98˚C, 30 sec at 60˚C, 3 min at 72˚C (2 min for nested PCR), and a final extension for 5 min at 72˚C.

PCR products (4 *μ*l) was stained with GelRed (Biotium), analyzed by electrophoresis through 1.0% agarose gel and photographed. The amplified fragments were purified using the NucleoSpin Gel and PCR clean-up kit (Macherey-Nagel), and direct sequencing was performed using the light run sequencing service of GATC Biotech (http://www.gatcbiotech.com/en/sanger-services/lightrun-sequencing.html). The BLAST (http://blast.ncbi.nlm.nih.gov/Blast.cgi) and BLAT (http://genome.ucsc.edu/cgi-bin/hgBlat) programs were used for computer analysis of sequence data.

### Real-time PCR

To quantify the expression of *CSF1* transcript 1 (NM_000757), *CSF1* transcript 4 (NM_172212), the new *CSF1* transcript described here, and the *CSF1-S100A10* fusion transcript, SYBR Green-based gene expression real-time PCR was used. The 20 *μ*l reaction volume contained 1X SsoAdvanced SYBR Green supermix (Bio-Rad), 0.5 *μ*M of each of the forward and reverse primers, and 2 *μ*l cDNA (40 ng equivalent of RNA). Four replicates of each sample were used to ensure statistical representativity. For *CSF1* transcript 1 the primer set was CSF1-2057F and CSF1-trans1-2123R, for *CSF1* transcript 4 the primer set was CSF1-2057F and CSF1-trans4-2120R, for the newly identified *CSF1* transcript 5 the primer set was CSF1-2057F and CSF1-transNew-Rq, and for the *CSF1-S100A10* fusion transcript the primer set was CSF1-2057F and S100A10-664R. To evaluate the efficiency of the real-time PCR reactions, standard curves were generated using serial dilutions of purified PCR products which were amplified with Premix Taq and the primer set described above in ‘PCR and 3′-RACE’. To generate standard curves seven 10-fold dilutions were prepared for each PCR product starting with 0.1 ng. The efficiency (E), correlation coefficient (R^2), slope and y-intercept are given in Table II.

Real-time PCR was run on CFX96 Touch^™^ Real-Time PCR Detection system (Bio-Rad). The thermal cycling included an initial step at 95˚C for 30 sec, followed by 40 cycles of 10 sec at 95˚C and 30 sec at 60˚C. Melting curve analysis at the end of the PCR was performed in order to confirm whether or not a single product was amplified and that no primer dimers interfered with the reaction. There was an initial denaturation step at 95˚C followed by temperature rising from 65 to 95˚C with 0.5˚C increment for 0.05 sec. The data were analyzed using the Bio-Rad CFX Manager Software (Bio-Rad).

For quantification of the expression of *CSF1* and *CSF1R* transcript two assays were performed which were supplied by Applied Biosystems. Assay Hs00174164_m1 was used for the expression of *CSF1*. This assay was specific for exon 4/5 boundary and detects all the four reported transcripts of *CSF1* (accession nos. NM_000757, NM_172210, NM_172211 and NM_172212). Assay Hs00911250_m1 was used for *CSF1R* end of the transcript and was specific for exon 20/21 boundary (NM_005211). The assay Hs99999901_s1 18S (Applied Biosystems) was used as endogenous control for relative gene expression quantification. Four replicates of each sample and endogenous control were again used to ensure statistical representativity. The 20 *μ*l reaction volume contained 1X TaqMan Universal Mix, 1X 20X TaqMan Gene Expression Mix and 2 *μ*l cDNA (40 ng equivalent of RNA). Real-time PCR was run on CFX96 Touch™ Real-Time PCR Detection system (Bio-Rad). The thermal cycling included an initial step at 50˚C for 2 min, followed by 10 min at 95˚C and 40 cycles of 15 sec at 95˚C and 1 min at 60˚C. The data were analyzed using the Bio-Rad CFX Manager Software (Bio-Rad).

## Results

The cytogenetic analysis of case 1 showed that there were two unrelated abnormal clones: 46,XX,dic(2;13)(q11;p11),der(11) t(2;11)(q11;p15)[3]/46,XX,r(11)[2]/46,XX[33]. The karyotypes of cases 2 and 3 were reported previously (11). Case 2 had the karyotype 46,XY,t(1;22)(p13;q12)[20], while the karyotype of case 3 was 46,XX,t(1;1)(q21;p11)[9]/47,XX,+7[2]/46,XX[14].

**Figure 1.**
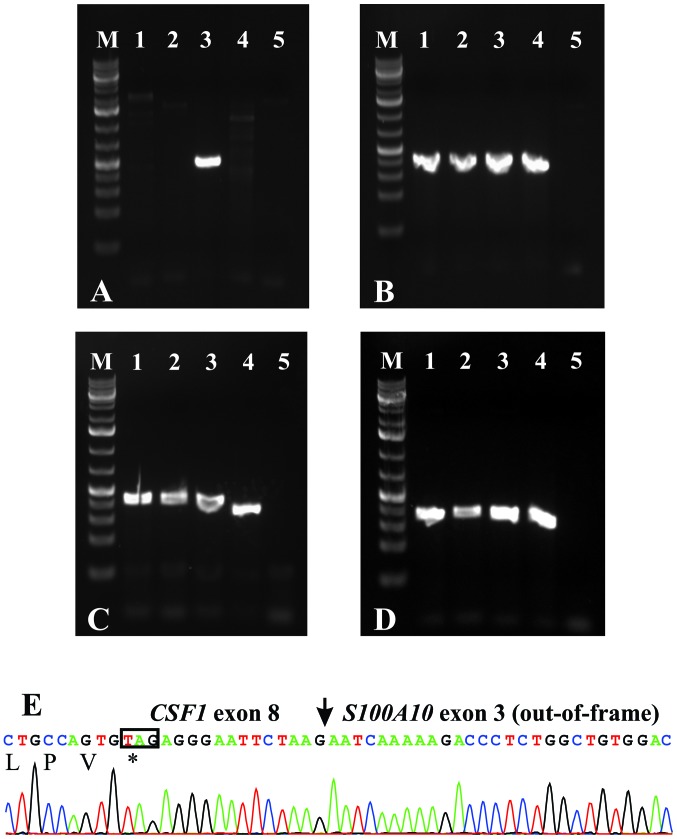
RT-PCR analysis of TSGCT. (A) cDNA fragment amplification using the primer set CSF1-1886F/S100A10-840R. (B) cDNA amplification of the wild-type *S100A10* using the primer set S100A10-555F/S100A10-840R. (C) cDNA amplification of the *CSF1* transcript 1 using the primer set CSF1-1886F/CSF1-2176R. (D) cDNA amplification of the *CSF1* transcript 4 using the primer set CSF1-1886F/CSF1-trans42130R. Lane M, 1 kb Plus DNA ladder (GeneRuler, Fermentas); lane 1, cDNA from case 1; lane 2, cDNA from case 2; lane 3, cDNA from case 3; lane 4, cDNA synthesized from Human Universal Reference Total RNA (Takara); lane 5, blank, no RNA in cDNA synthesis. (E) Partial sequence chromatogram of the cDNA fragment amplified with the primer set CSF1-1886F/S100A10-840R showing the fusion of exon 8 of *CSF1* with exon 3 of *S100A10*. The stop codon TAG is in the box.

Using the FusionMap on the raw sequencing data obtained from the Norwegian Sequencing Centre, the *CSF1-S100A10* fusion transcript, ranked 1st with 599 seed counts, was found in case 3, which carried the translocation t(1;1)(q21;p11), whereas no *CSF1* fusion transcript was found in the other two tumors. Because *CSF1* and *S100A10* map to chromosome bands 1p13.3 and 1q21.3, respectively, we decided to study the *CSF1-S100A10* fusion transcript. RT-PCR with the CSF1-1886F/S100A10-840R primer combination amplified a single cDNA fragment in case 3, but not in cases 1 and 2 (Fig. 1A). The wild-type *S100A10* cDNA, the *CSF1* transcript 1 (NM_000757) and the *CSF1* transcript 4 (NM_172212) were amplified in all cases (Fig. 1B–D). Sequencing of the fragment amplified with the CSF1-1886F/S100A10-840R primer combination showed that exon 8 of *CSF1* (nt 2091 in sequence with accession no. NM_000757 version 5) was fused to exon 3 of *S100A10* (nt 641 in sequence with accession no. NM_002966 version 2) (Fig. 1E).

**Figure 2.**
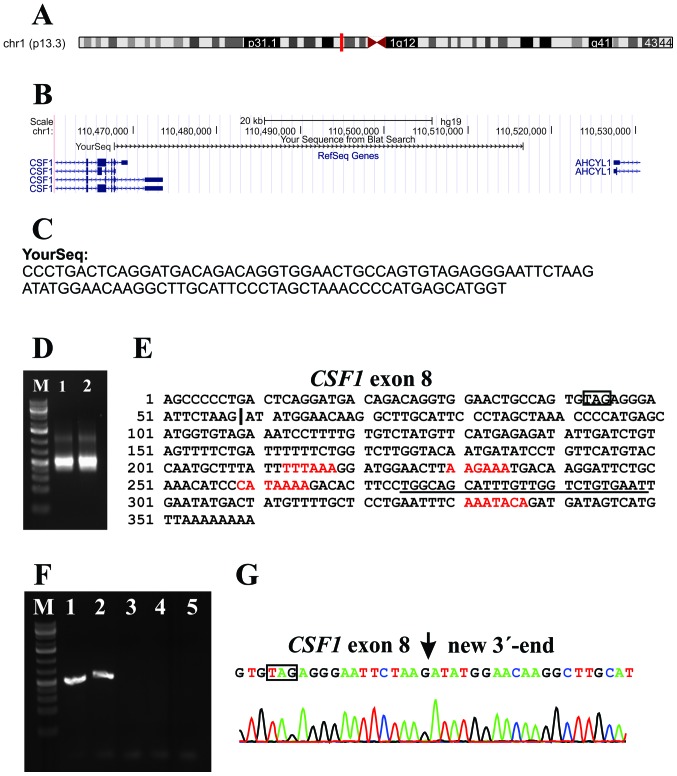
Analysis of the new *CSF1* transcript 5 in TSGCT. (A) Localization of the BLAT results on the chromosome 1 as it is obtained from genome browser. (B) Results of the BLAT search with one of the reads (YourSeq) which contained the last 20 nt of exon 8 of *CSF1* (agtgtagagggaattctaag; nt 2081–2091 in sequence with accession no. NM_000757) and extracted from the raw sequencing data. (C) The sequence of the YourSeq which was used for BLAT search. (D) 3′-RACE on the cases 1 and 2 amplified a single cDNA fragment. (E) Sequence of the 3′-RACE-amplified cDNA fragment. The stop codon TAG is in box. The vertical line is the junction between exon 8 of *CSF1* and the new sequence on 1p13 which is 41 kb downstream from the currently known *CSF1* locus. The red letters are the polyadenylation signals. The primer CSF1-3end-R1out is underlined. (F) RT-PCR amplification using CSF1-1886F/CSF1-3 end-R1out primer combination. In case 1 (lane 1) and case 2 (lane 2), a single cDNA fragment is amplified. In case 3 (lane 3), the control cDNA (lane 4) and blank (no RNA in cDNA) (lane 5), no fragments are amplified. M is 1 kb Plus DNA ladder (GeneRuler, Fermentas). (G) Partial sequence chromatogram of the cDNA fragment amplified with primers CSF1-1886F/CSF1-3end-R1out showing the fusion of exon 8 of *CSF1* with the new 3′-end.

**Figure 3.**
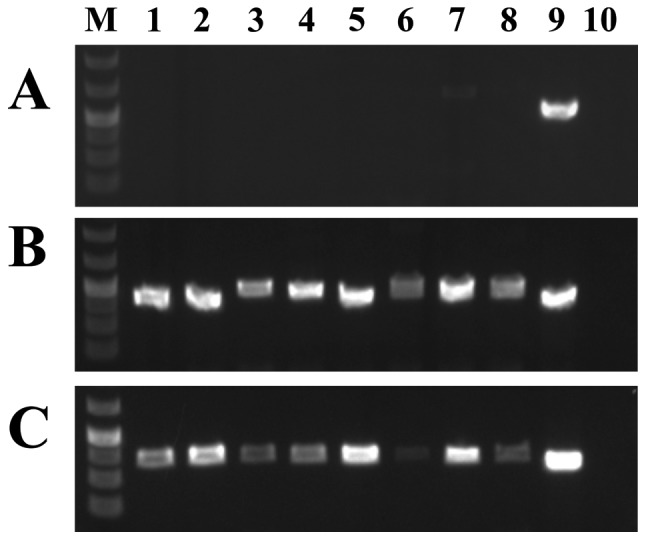
Expression analysis of the various *CSF1* transcripts in a cDNA panel of eight cell lines. (A) Expression of *CSF1* transcript 5. (B) Expression of *CSF1* transcript 1. (C) Expression of *CSF1* transcript 4. Expression analysis was performed using the human cell line MTC cDNA panel (Clontech). Lane 1, Ad5 cell line; lane 2, SKOV-3; lane 3, Saos2; lane 4, A431; lane 5, Du145; lane 6, H1299; lane 7, HeLa; lane 8, MCF7; lane 9, case 1 which was used as positive control; lane 10, blank (no RNA in cDNA). Lane M is 1 kb Plus DNA ladder (GeneRuler, Fermentas).

Because Cupp *et al* (23) found expression of *CSF1* in all studied TSGCT, including those that lacked the *CSF1* translocation, we decided to investigate further the expression of *CSF1* in cases 1 and 2 which did not have *CSF1*-fusion transcripts. No other fusion transcripts or genes were examined. We retrieved reads from the raw sequencing data which contained the last 20 nt of exon 8 of *CSF1* (agtgtagag ggaattctaag; nt 2072–2091 in sequences with accession nos. NM_000757 and NM_172212) from both cases 1 and 2 and performed a database search by applying the BLAST and BLAT algorithms (Fig. 2A and B). The analyses showed that exon 8 of *CSF1* was fused to a sequence with features, according to BLAST, ‘48731 bp at 5′ side: macrophage colony-stimulating factor 1 isoform a precursor and 11081 bp at 3′ side: putative adenosylhomocysteinase 2 isoform a’ (Fig. 2C). 3′-RACE amplified a single fragment in cases 1 and 2 (Fig. 2C). Sanger sequence analysis of the amplified fragment verified the data obtained by RNA-Seq, i.e., the fusion of *CSF1* exon 8 with the new sequence, and showed that the latter had a poly-adenylation signal, AAATACA, close to the polyA tail (Fig. 2E). Three other poly-adenylation signals were found in this sequence (Fig. 2E). PCR with the CSF1-1886F/CSF1-3end-R1out primer combination (Table I) amplified a single cDNA fragment in cases 1 and 2 (Fig. 2F). Sequence analysis of these fragments verified the results obtained by RNA-Seq and 3′-RACE (Fig. 2G). Expression analysis of 8 cell lines showed that none of them expressed the new CSF1 transcript whereas both transcripts 1 and 4 were expressed (Fig. 3). Because the new non-genic sequence fused to exon 8 of *CSF1* is 48 kb downstream of the currently known *CSF1* locus, we consider it as a new alternative exon and we call this novel sequence *CSF1* transcript 5.

**Figure 4.**
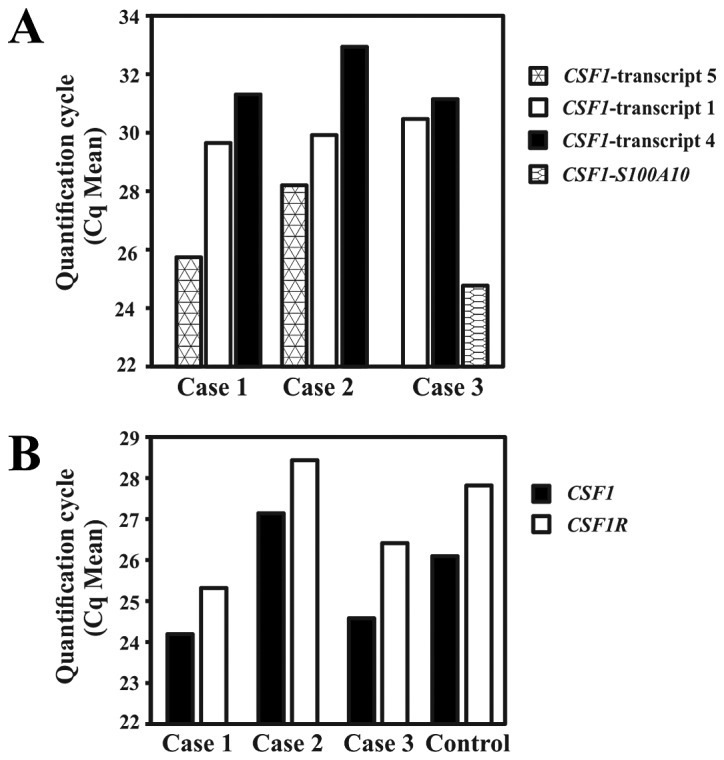
Real-time PCR to quantify the transcripts 1, 4 and 5 of *CSF1*, *CSF1-S100A10* and *CSF1R* in TSGCT. (A) Quantification of the *CSF1* transcripts 5, 1 and 4 in cases 1 and 2, and *CSF1-S100A10*, and *CSF1* transcripts 1 and 4 in case 3. (B) Quantification of the expression of the *CSF1* gene (all transcripts together) and its receptor *CSF1R*. The expression is presented as quantification cycle (Cq mean).

Real-time PCR to quantify the expression of the *CSF1* transcripts and *CSF1-S100A10* showed that in cases 1 and 2, the new transcript 5 was the most highly expressed followed by transcripts 1 and 4 (Fig. 4A). In case 1, the mean quantification cycle (Cq mean) was 25.74, 29.65 and 31.31 for transcript 5, transcript 1 and transcript 4, respectively. In case 2, the respective values for Cq mean were 28.2, 29.92, and 32.94. In case 3, the highest expression was observed for the fusion *CSF1-S100A10* transcript (Cq mean = 24.77) followed by *CSF1* transcripts 1 (Cq mean = 30.47) and 4 (Cq mean = 31.15).

Real-time PCR to quantify the expression of *CSF1* (all transcripts) and *CSF1R* showed that *CSF1* was slightly higher expressed than *CSF1R* in all cases, including the control Human Universal Reference Total RNA. The Cq means for *CSF1/CSF1R* were 24.19/25.32, 27.1/28.44, 24.58/26.41 and 26.09/27.82 for cases 1, 2, 3 and the control, respectively (Fig. 3B).

## Discussion

We have identified a novel *CSF1-S100A10* fusion gene in a TSGCT carrying the translocation t(1;1)(q21;p11). In this fusion gene, the part of *CSF1* coding for the CSF1 protein (exons 1–8 in sequences with accession nos. NM_000757 and NM_172212) is fused to the 3′-part of *S100A10*. Since the stop codon TAG of *CSF1* is present in the fusion gene (Fig. 1C), the consequence of the *CSF1-S100A10* seems to be replacement of the 3′-untranslated region (UTR) of *CSF1* (exon 9; nt 2092–4234 in sequence with accession no. NM_000757 or nt 2092–2772 in NM_172212) by the 3′-end of *S100A10* (exon 3; nt 641–1055 in sequence with accession no. NM_002966).

The *CSF1-S100A10* fusion gene is reminiscent of the *HMGA2*-fusions in benign connective tissue tumors. Chromosomal rearrangements involving 12q13–15 and targeting *HMGA2* result in mostly out-of-frame fusion genes in which a stop codon is encountered quickly so that only a few amino acids are added to the AT-hook of HMGA2 (25). The fusion also has another result, namely the removal of the 3′-UTR of *HMGA2* which contains multiple let-7 binding sites. Let-7 miRNA might act as a repressor of *HMGA2* and miRNA-directed repression of an oncogene could provide a mechanism of tumorigenesis (25).

*S100A10* has been reported as the 3′-partner gene in the *HDGF/S100A10* fusion gene which was found in the UACC-812 breast cancer cell line (26). *S100A10* codes for a member of the S100 family of proteins containing 2 EF-hand calcium-binding motifs. S100 proteins are located in the cytoplasm and/or nucleus of a wide range of cells and are involved in the regulation of a number of cellular processes such as cell cycle progression and differentiation. S100 genes include at least 13 members which are located as a cluster in chromosome band 1q21 (http://www.ncbi.nlm.nih.gov/gene/6281). S100A10 plays a role in oncogenesis by regulating the plasmin proteolytic activity of cancer cells and by regulating the migration of macrophages to the tumor site (27).

In the other two TSGCT we were able to examine, no fusions of *CSF1* with known genes were found. Instead, a novel fusion of the coding region of *CSF1* with a roughly 300 bp sequence located 48 kb downstream of the CSF1 locus was detected, identical in both cases. Based on the location of the new sequence which is fused to exon 8 of *CSF1* (48 kb downstream of the currently known *CSF1* locus), we call this novel sequence *CSF1* transcript 5. However, the possibility that this transcript is a product of the chromosome aberrations of case 1 and the chromosome translocation t(1;22) found in case 2 cannot be ruled out. Similar to the *CSF1-S100A10* fusion gene, the novel *CSF1* transcript 5 has the 3′-UTR of *CSF1* (exon 9; nt 2092-4234 in sequence with accession no. NM_000757 or nt 2092–2772 in NM_172212) replaced by a new exon located 48 kb downstream of *CSF1* and 11 kb upstream of *AHCYL1* (adenosylhomocysteinase-like 1) (Fig. 2C). The novel *CSF1* transcript 5 was not found in the Human Universal Reference Total RNA which is a mixture of total RNAs from adult human tissues and a panel of 8 well known cell lines (Figs. 2F and 3). At the moment, both its frequency and its specificity for TSGCT are unknown.

Cupp *et al* (23) described two groups of TSGCT/PVNS defined by CSF1 biology. The first group had *CSF1* rearrangements and high levels of *CSF1* RNA expression. In the second group of TSGCT, there were no *CSF1* rearrangements as determined by FISH, but the same characteristic *CSF1* RNA and CSF1 protein expression pattern was nevertheless present. Thus, an alternative mechanism must exist leading to *CSF1* upregulation in this tumor subset. An altered CSF1-CSF1R signaling pathway still appears to be a critical tumorigenic event in these TSGCT/PVNS, but the putative translocation or mutation seems to involve a gene whose product leads to upregulation of *CSF1*. An alternative mechanism for at least some cases could be the novel *CSF1* transcript presented here.

Although we studied only 3 TSGCT, a common pathogenetic theme is discernible shared by the *CSF1-S100A10* fusion gene and the novel *CSF1* transcript: the replacement of the 3′-UTR of *CSF1* with new sequences. This replacement results in expression of the protein-coding part of *CSF1*. Thus, the reported t(1;2) found in TSGCT might not bring *CSF1* under control of the promoter of *COL6A3* as has been proposed (20,21) but might instead result in the replacement of the 3′-UTR of *CSF1* with sequences from *COL6A3* in a similar way to what we describe here.

While there is no information on the 3′-UTR of *CSF1* transcript 4 (sequence with accession no. NM_172212), there is ample information on the 3′-UTR region of *CSF1* transcript 1 (sequence with accession no. NM_000757). The exon 9 of *CSF1* mRNA (accession no. NM-000575) contains microRNA targets (miRNA), a non-canonical G-quadruplex, and AU-rich elements (AREs) which control the expression of *CSF1* (28–31). It has at least 14 miRNA sites (30) and it was shown that both miRNA-128 and miRNA-152 downregulate the expression of *CSF1* and its protein (30). Two other miRNAs, miR-130a and miR-301a, in the presence of a nucleolin also downregulate the expression of *CSF1* (29). AREs are known to dictate mRNA (32). For the AREs of CSF1, glyceraldehyde-3-phosphate dehydrogenase was shown to bind to the AU-rich elements and regulates the stability and decay of *CSF1* mRNA (28,31). Recently, Woo *et al* (29) showed that 3′-UTR of *CSF1* contains a non-canonical G-quadruplex which is involved in the post-transcriptional regulation of *CSF1* and that nucleolin is the interacting protein. In the same study, using a luciferase reporter system fused to *CSF1* mRNA 3′-UTR, they showed that the disruption of the miRNA target region, G-quadruplex, and AREs together dramatically increased reporter RNA levels, suggesting important roles for these *cis*-acting regulatory elements in the downregulation of *CSF1* mRNA.

The CSF1-CSF1R signaling pathway seems to be critically involved in TSGCT/PVNS tumorigenesis something that has led to the trial of alternative therapies based on tyrosine kinase inhibition with imatinib mesylate. In addition to its inhibitory activity on BCR-ABL, KIT, and PDGFRA, imatinib mesylate has been reported to block CSF1R activation at therapeutic concentrations (33). After the first report of complete response obtained with imatinib in a single patient with TSGCT (34), imatinib mesylate has been tried in several patients who were affected with locally advanced and/or metastatic TSGCT, and the response has been promising (34–36). Also for this reason a better understanding of the mechanisms of *CSF1* activation in TSGCT and the role played by the CSF1-CSF1R signaling pathway are important. More tumors must be studied in order to determine the frequency and specificity of the new transcript as well as the possible ubiquity of the replacement of the 3′-UTR of *CSF1* in aberrations targeting the *CSF1* gene.

## Figures and Tables

**Table I.** Primers used for PCR amplifications and sequencing.

Oligo name	Sequence (5′→3′)
CSF1-1886F	GCA GCT CCA GGA GTC TGT CTT CCA C
CSF1-2006F	GGA TTC TCC CTT GGA GCA ACC AGA
CSF1-2057F	ACA GGT GGA ACT GCC AGT GTA GA
CSF1-2176R	AGC TCT GGT GGA GGG CAG ACC A
CSF1-3end-R1out	ATT CAC AGA CCA ACA AAT GCT GCC A
CSF1-trans4-2130R	GCT GGG CGT CAC ATT TTC AGA GG
CSF1-3end-R2out	TGG GAT GTT TGC AGA ATC CTT GTC A
CSF1-trans1-2123R	GTG GAC GCC CCA TAA TGT CTC
CSF1-trans4-2120R	CAC ATT TTC AGA GGG ACA TTG ACA
CSF1-transNew-Rq	ACA CCA TGC TCA TGG GGT TTA G
S100A10-555F	TTC ACA AAT TCG CTG GGG ATA AAG G
S100A10-664R	TCC AGG TCC TTC ATT ATT TTG TCC
S100A10-809R	TAT CAG GGA GGA GCG AAC TGC TCA T
S100A10-840R	GAT TCC TTA AGC GAC CCT TTG GGA C
A3RNV-RACE	ATC GTT GAG ACT CGT ACC AGC AGA GTC ACG AGA GAG ACT ACA CGG TAC TGG TTT TTT TTT TTT TTT
A3R-1New	TCG TTG AGA CTC GTA CCA GCA GAG TCA C
A3R-2New	GAG TCA CGA GAG AGA CTA CAC GGT ACT GGT T

**Table II.** Standard curve analyses of the real-time PCR for the quantification of the expression of *CSF1* transcripts 1, 4 and 5, and *CSF1-S100A10*.

Template (purified PCR) product	Gene-transcript	Accession no.	Primer set for real-time PCR	Efficiency (E %)	Correlation coefficient (R^2)	Slope	y-intercept
CSF1-1886F/CSF1-2176R	*CSF1*-transcript 1	NM_000757 version 5	CSF1-2057F/CSF1-trans1-2123R	107.1	0.998	3.164	29.532
CSF1-1886F/CSF1-trans4-2130R	*CSF1*-transcript 4	NM_172212 version 2	CSF1-2057F/CSF1-trans4-2120R	98.3	0.997	3.364	29.690
CSF1-1886F/CSF1-3end-R1out	*CSF1*-transcript 5	-	CSF1-2057F/CSF1-transNew-Rq	97.6	0.997	3.380	30.662
CSF1-1886F/S100A10-840R	*CSF1-S100A10*	-	CSF1-2057F/S100A10-664R	110.1	0.991	3.102	29.282
